# Preventing and treating childhood obesity by sleeping better: a systematic review

**DOI:** 10.3389/fendo.2024.1426021

**Published:** 2024-09-19

**Authors:** Debora Porri, Giovanni Luppino, Tommaso Aversa, Domenico Corica, Mariella Valenzise, Maria Francesca Messina, Giorgia Pepe, Letteria Anna Morabito, Elisa La Rosa, Cecilia Lugarà, Tiziana Abbate, Roberto Coco, Francesca Franchina, Aurora Lanzafame, Fabio Toscano, Alessandra Li Pomi, Paola Cavallaro, Malgorzata Gabriela Wasniewska

**Affiliations:** ^1^ Department of Human Pathology of Adulthood and Childhood, University of Messina, Messina, Italy; ^2^ Pediatric Unit "G. Martino", University Hospital, Messina, Italy

**Keywords:** childhood obesity, sleep, sleep hygiene, lifestyle, childhood obesity prevention, childhood obesity treatment

## Abstract

**Background:**

Childhood obesity represents a major public health issue worldwide. Evidence showed the need to implement prevention strategies mainly focused on lifestyle habits. Sleep hygiene is a variable of great interest and this review systematically examined the effects of sleep duration in increasing childhood obesity risk

**Methods:**

A systematic literature review was conducted from December 2023 to February 2024. Study selection and data extraction procedures were performed in accordance with Reporting Items for Systematic Reviews and Meta-Analyses (PRISMA) Guidelines and Statement, and risk of publication bias was assessed by the Effective Public Health Practice Project Quality Assessment Tool for Quantitative Studies.

**Results:**

Original works in English were eligible for review and eleven studies that met the inclusion criteria were included. Studies collected were heterogeneous in terms of duration, sample characteristics, hours of sleep manipulation, anthropometric and hematological parameters collected, therefore it was not possible to perform a meta-analysis. A narrative synthesis of the reported evidence highlighted the impact of sleep duration above all on food intake, eating habits and hormone levels and consequently on the risk of childhood obesity development.

**Conclusion:**

This finding suggests the need to consider sleep hygiene as a modifiable lifestyle habit like diet and physical activity, in order to early prevent childhood obesity. Poor sleep hygiene can significantly contribute to weight gain and exacerbation of metabolic disorders linked to childhood obesity. Although more rigorous studies are needed, clinicians need to be aware of the role of sleep hygiene in reducing childhood obesity risk.

## 1 Introduction

Childhood obesity (CO) is considered a growing pandemic due to its steadily increasing incidence, representing a serious health problem worldwide (1). CO has become an undeniable public health crisis, especially with the effects of the pandemic and national blocking measures that have led children and adolescents to lead more sedentary lifestyles (1, 2). Children with obesity or overweight have metabolic and cardiovascular risk factors that can turn out as early as childhood and adolescence, and lead to a deterioration in both quality and perspective of life and are linked with increased mortality during adulthood. Therefore, in the management of child health, obesity prevention and treatment are one of the priorities (1, 3).

The etiology of CO is multifactorial. Poor lifestyles are implicated in the development of overweight or obesity and include increased screen time with less physical activity and the ingestion of high-calorie foods (3). The COVID-19 pandemic has significantly disrupted daily practice, resulting in decreased physical activity levels, increased sedentary time, and disrupted sleep pattern, significantly contributing to an increased risk of CO. In addition, daily lifestyle habits play an important role in determining the equilibrium of the subjects’ circadian rhythms. Clinical and epidemiological studies have demonstrated the role of altered circadian rhythms in the development of obesity, as revealed by studies of shift workers who have an increased risk of developing obesity-related complications (4–6). Several animal and human studies have shown that sleep duration can influence metabolic health and this hypothesis is based on the interaction between biological rhythms, eating patterns, meal time and its influence on metabolic health (7–9). The circadian system is characterized by a master clock located in the suprachiasmatic nuclei (SCN) of the hypothalamus and other neurological centers in the brain. In addition to the SCN, several peripheral tissues have cells with molecular clock activity that contribute to local tissue activity during day and night (10).

The central circadian clock regulates metabolism through hormonal mechanisms, such as the release of melatonin and cortisol, and indirectly through neuronal pathways by programming the sense of hunger and sleep. The disruptions of circadian mechanisms may arise from exogenous factors, i.e. shift work, nocturnal light exposure, and social jet lag, and from endogenous factors, like genetic variants and sleep disorders or alterations of timing to eat (11). Particularly, recent research has identified associations between sleep hygiene in terms of duration, quality and timing and risk of overweight/obesity both in children and adults (12). Food intake at several times of the day and the entity/quality of sleep may influence weight and risk of obesity (13, 14). A new frontier in the treatment and primarily in the prevention of obesity is knowledge of sleep hygiene and child-specific chronotypes, modulated by modifiable lifestyle habits such as eating habits but also sleep and physical activity. Childhood represents a delicate age in which lifestyle, food preferences and sleep patterns develop. It is the time window with the greatest preventive potential and in this period, it is essential to have a correct lifestyle and learn not only what and how much but also when to eat and sleep.

## 2 Methods

This systematic review has been conducted following the Preferred Reporting Items for Systematic Reviews and MetaAnalyses (PRISMA) guidelines (15) recommending to present a full electronic search strategy for at least 1 major database PubMed were searched from 2014 to 2024 using the following structured search strings: (pediatric obesity) OR (pediatric overweight)) OR (childhood obesity)) OR (childhood overweight)) OR (obesity in children)) OR (overweight in children)) OR (overweight risk)) OR (obesity risk)) OR (childhood obesity treatment*)) OR (childhood obesity prevention)) OR (pediatric obesity treatment*)) OR (pediatric overweight prevention)) OR (diet)) OR (nutrition)) AND (meal time)) OR (meal frequency)) OR (mealtime)) OR (late eater*)) OR (late eating)) OR (meal skipper*)) OR (clock* gene)) OR (circadian clock)) OR (circadian rhythm*)) OR (sleep deprivation)) OR (sleep duration)) OR (sleep alteration).

### 2.1 Eligibility criteria

All studies were assessed according to the following inclusion and exclusion criteria:

#### 2.1.1 Participants

Eligible subjects were children ages ranging from 2 to 18 years old. Children with essential obesity/overweight (as defined in the selected studies) are also included.

#### 2.1.2 Intervention

Studies with an approach covering observational or interventional on sleep behavior and its influence on lifestyle habits were included.

#### 2.1.3 Comparison

Different study designs, randomized controlled trials, case–control studies and cross-sectional studies, were included in this review.

#### 2.1.4 Outcome

The outcome of this systematic review was to evaluate the role of sleep hygiene which could lead to overweight/obesity development by a disruption of circadian rhythms and/or lifestyle habits in children and adolescents. Secondary outcome was to evaluate the impact of lifestyle changes on weight loss, expressed as Body Mass Index (BMI), BMI z score (BMIz), and BMI Standard Deviation Score (BMISDS) as a possible confounding factor.

#### 2.1.5 Exclusion criteria

The comprehensive research strategy retrieved studies that were unlinked to the aim of this systematic review and were subsequently excluded. Narrative reviews, systematic reviews case reports, case series, letters, comments, and articles that did not correspond to the outcome of this review were excluded, as well as studies without a full text accessible in English.

#### 2.1.6 Selection process

DP and MGW conceptualized the comprehensive search strategy. Titles and abstracts were screened by ELR, CL, TA, RC, FF, AL, FT and ALP for inclusion. DP, GL, TA, DC, MV and MFM structured the main text. DP, GP and LAM designed the tables and figures. PC provided the language editing. Reference lists of articles were checked to identify any other study appropriate for inclusion. Studies assessed as eligible, potentially eligible or unclear, were retrieved in full text. Any uncertainty concerning the inclusion of specific studies was resolved by discussing with MGW. Last search date 31/03/2024.

#### 2.1.7 Data extraction and synthesis

The final studies included in the review were described in the main text and in standardized tables. Due to the heterogeneity of study population characteristics (age, ponderal status) and lifestyle variables assessed, we could not perform a meta-analysis but a narrative summary of the findings was conducted.

### 2.2 Quality assessment and risk of bias

Study quality was assessed in duplicate using a designed appraisal tool, the Effective Public Health Practice Project Quality Assessment Tool for Quantitative Studies, a tool for systematic reviews which evaluates randomized and non-randomized intervention studies (16). Individual component quality rankings and the risk of bias measures are included in **Supplementary Table 1**. Studies’ components and overall quality ratings were scored as “strong,” “moderate” or “weak” according to the tool’s instructions (16, 17).

## 3 Results and discussion

### 3.1 Overview

**Figure 1:**
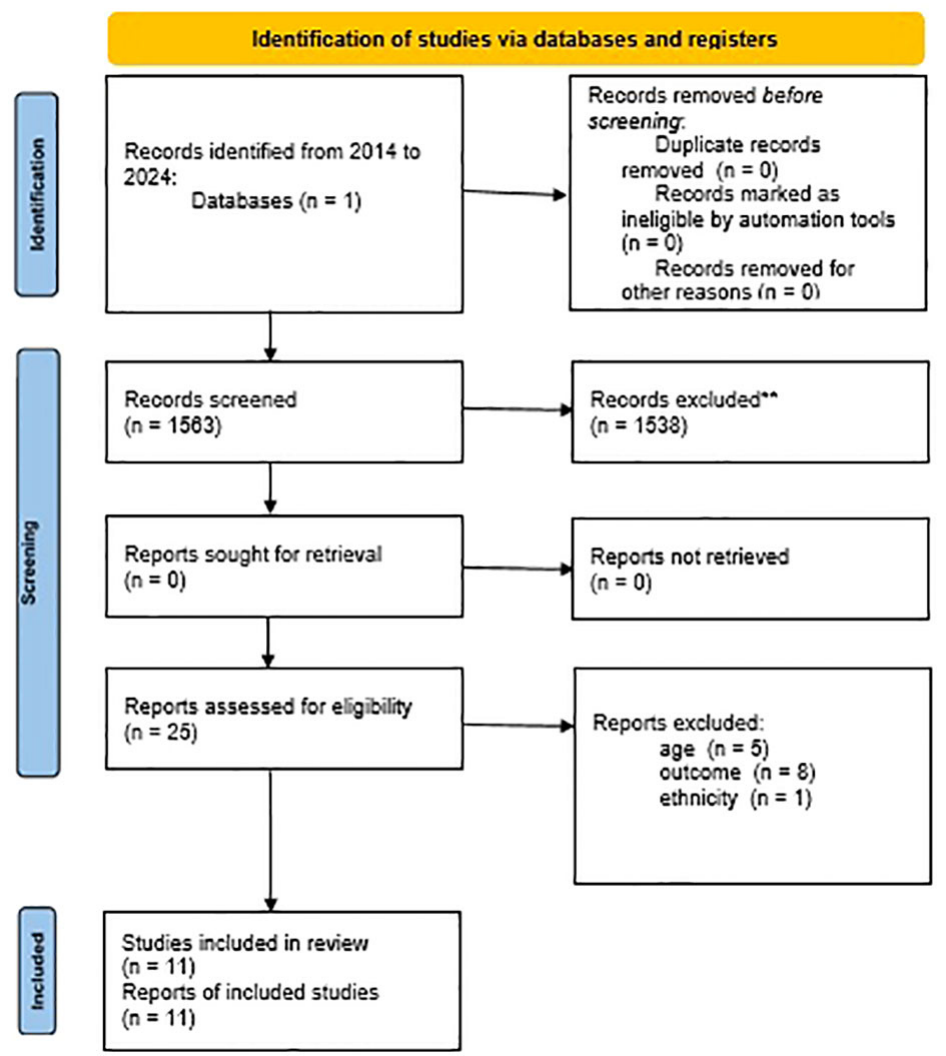
Prisma Flow Diagram.

A flowchart summarizing the study selection procedure is presented in **Figure 1**. Electronic searches returned 1563 records. 25 studies were retained after screening the titles and abstract while 14 studies were further excluded after reading through full texts. Of the 14 excluded records, 6 referred to the wrong population (5 didn’t include pediatric age and 1 was excluded due to participant ethnicity) while 8 studies referred to a different outcome than the one investigated. Finally, only 11 eligible studies were included in this systematic review. Notably, two studies didn’t evaluate sleep duration or provide sleep manipulation, but we decided to include them as they take into consideration some aspects that can be significantly influenced by sleeping habits, as discussed below.

### 3.2 Study characteristics

Characteristics of selected studies are reported in **Table 1**. All studies have been published between 2013 and 2024. Three studies were conducted in Europe (18–20) while one was set in the United Kingdom (21), two study took place in the US (22, 23) while one study was conducted in Canada (24) one study was conducted in Mexico (25), two in New England (26, 27) and one study took place in the New Zealand (28). Considering participant’s age, only one study (18) included children in the preschool age (3-8 years old) while other studies evaluated participants in adolescence and preadolescent age with age ranging from 6 to 17 years old (19–24, 26–28). One study also included 18 years old subjects (25). We collected four studies with a sample size over 1000 participants (19–21, 24), three studies enrolled a range from 100 to 726 subjects (18, 25, 28) while four studies had a sample size under 100 participants (22, 23, 26, 27). Only one study (25) performed an obesity treatment while in the other studies authors observed the effect of lifestyle variables (sleep duration, meal time, physical activity) on anthropometric parameters and/or hormone levels and obesity markers. Data collection for each studies was described in **Table 2**.

**Table 1:** Characteristics of selected studies.

Reference	Study design	Trial registration (if availabe)	Simple size (Participants)	Sample age	Country(es)	Setting(s)	Intervention	Variables assessed (Comparison)	Outcome	Follow up length	Statistical model
Jaeger V. et al, 2022 (18)	Observational study	NCT0033868	729	3 to 8 years - old	Germany, Spain, Italy, Poland and Belgium	Home and outpatients clinic	/	Weight; height; BMI; BMI-Z. Numbers of Eating Occasions; Caloric intake.	To analyse the effect of the distribution of energy and nutrients in different meals on the BMI z-score during childhood.	7 years	Linear regression analysis and linear mixed effects models
Bodega P. et al, 2023- (19)	Clinical trial	NCT03504059	1183	11-14 years old	Spain	Secondary schools	/	Weight and percentage of body fat, height, waist circumference, BMIz. Food Frequency Questionnarie. Step counts: Seep time and leisure. Screen time	To identify and analyze the relationship between lifestyle patterns and adiposity in early adolescents	2-year and 4- year follow-up, (complete baseline information obtained in 2017)	Cluster analysis and Generalized mixed models
Martínez- Gómez J, A et al 2023 (20)	Cluster- randomized controlled intervention trial	NCT03504059	1216	12-16 years old	Spain	Hospital/ home	Characterized sleep duration and its cross- sectional and longitudinal associations with adiposity markers in adolescence.	Fat mass index, height, waist circumference, BMI, waist to hip ratio. Hours of sleep	To examine the cross-sectional and longitudinal associations between sleep duration and a panel of adiposity markers during adolescence.	4 years	χ2 test with the Cochran–Mantel– Haenszel extension test (for variables with ordered categories); ANOVA (for continuous variables), multilevel linear mixed-effects models. Generalized linear with Poisson models (for associations between sleep duration groups and overweight/obesity prevalence)
Donin et al., 2014 (21)	Cross-sectional (observational) study	/	4116	9-10 years old	UK	Outpatient clinic	/	Breakfast consumption (frequency and content); measurements of body composition, fasting blood samples of blood lipids, insulin, glucose, and glycated haemoglobin	/	/	Multilevel linear regression models
Simon SL et al. 2015 (22)	Randomized cross-over sleep restriction- extension paradigm.	/	31	14-17 years old	USA	Home/Hospital	All subjects were randomly assigned to 5 consecutive nights of sleep restriction (6.5 hours in bed) versus healthy sleep duration ( 10 hours in bed)	Hours of sleep. Food-appeal. Hunger rating	To examine the effect of experimental sleep restriction on subjective hunger and perceived appeal of sweet/dessert foods versus other foods.	3 weeks	General linear modeling
Beebe D.W. et al, 2015 (23)	Randomized controlled trial	/	67	14 to 17- years-old	Ohio, Massachusetts, Colorado	Home and outpatients clinic	Change bedtimes to create five- night periods of sleep restriction (6.5 hours in bed)	Hours of sleep. Caloric intake	To test if sleep extension by an earlier bedtime affects differently the food intake of adolescents accustomed to a later sleep phase ("night owls") compared to an earlier sleep phase ("larks")	three week	Linear mixed effects models
Carson V, et al., 2016 (24)	Randomized controlled trial	/	5217	6 to 17 years old	Canada	Home	/	BMI, BMIz, systolic and diastolic blood pressure, and behavioural strength and difficulties. Sedentary time, light- intensity physical activity and moderate- to vigorous-intensity physical activity	To examine the relationships between movement behaviours (sleep duration, sedentary time, physical activity) and health indicators	7 days	Linear regression models
Moreno- Frías C et al. 2020 (25)	Randomized controlled trial	/	52	14 - 18 years old	Mexico	Home and outpatients clinic	Adolescents were subjected to a diet with 500 calories restriction, randomly allocated to groups without and with sleep extension (increasing 5 minutes each night, Monday through Friday to reach the target of 1 hour)	Weight, height, and waist circumferences, fasting glucose, lipid profile, and creatinine. Serum interleukin 6, tumor necrosis factor a, leptin, and insulin. Cortisol and 6- sulfatoxymelatonin. Hours of sleep. Caloric intake	To identify the differences about of antropometric and laboratory data between both groups with and without sleep extantion.	4 weeks.	Student's t-test or Wilcoxon test, Paired Student's t-test for dependent samples.
Hart C.. et al, 2013 (26)	Randomized controlled trial	NCT01030107	39	8-11 years old	Southeastern New England	Home	Children were randomized to either increase or decrease their time in bed by 1.5 hours per night for 1 week	Height,weight, BMIz, level of leptin, ghrelin. Hours of sleep. Caloric Intake.	To determine whether changes in school-age children’s sleep duration are associated with changes in eating behaviors, appetite- regulating hormones, and measured weight	3 weeks	Repeated measures analysis of variance and paired sample t test
Hart C. et al., 2022 (27)	Randomized controlled trial	/	39	8-11 years old	Southeastern New England	Home and outpatient clinic	Children were randomize to increase or not their time in bed (TIB) by 1.5 hours/night for 1 week or decrease TIB by 1.5 hours/night for 1 week with the goal of creating a difference.	Weight; height. Hours of sleep. Caloric intake.	To examine experimental changes in children’s sleep period on reported caloric intake from energy-dense snack foods and sugar- sweetened beverages	three-week	Multiple linear regressions estimated by Generalized Estimating Equations (GEE). Paired t-tests and McNemar’s tests.
Morrison S, J, et al. 2023 (28)	Randomized, crossover study	CTRN1261800 1671257.	105	8-12 years old	New Zealand	home environ- ment	All participants were asked to go to bed 1 h later than their usual bedtime each night for 1 wk (sleep restriction) and 1 h earlier than their usual bedtime each night for 1 wk (sleep extension), separated by a 1- wk washout	Dietary intake (2 24-h recalls/wk), eating behaviors	To determine how changes in sleep influence energy intake and eating behavior	/	Data were analyzed according to the intention to treat and per protocol

/ means that data are not available.

**Table 2:** Data collection.

Reference	Anthropometric parameters						Blood sample										Hormone levels				Dietary habits				Sleeping habits		Physical activity	
	Blood pressure	Weight, Height, BMI	WC	WhTr	BIA	Skinfold thickness	Glucose	Hba1c	insulin	HOMA index	Lipid profile (Total cholesterol, LDL, HDL)	Triglycerides	PCR	Creatinine	IL-6	TNF- a	leptin	ghrelin	cortisol	6- sulfatoxymelatonin	24h- recall	FFQ	Food appealing	Food reinforce-men t	Actigraphy	Sleep diary	Screen time	Step count
**Bodega P. et al, 2023** (19)		✓	✓	✓	✓																	✓			✓		✓	✓
**Martin- Gomez J et al, 2023** (20)																									✓			✓
**Morrison S et al 2023** (28)							✓		✓	✓	✓	✓			✓	✓			✓	✓	✓	✓			✓			
**Jaeger V, et al 2022** (18)		✓																			✓							
**Hart C. et al. 2022** (27)		✓																			✓	✓						
**Moreno- Frías C et al. 2020** (25)		✓					✓		✓		✓			✓	✓	✓	✓		✓	✓	✓					✓		
**Carson V, et al., 2016** (24)	✓	✓	✓						✓		✓	✓	✓													✓		✓
**Simon SL et al, 2015 (** 22 **);**																					✓		✓		✓	✓		
**Beebe D.W. et al, 2015** (23)																					✓				✓			
**Donini et al, 2014** (21)	✓	✓	✓		✓	✓	✓	✓	✓		✓	✓	✓															
**Hart C. et al, 2013** (26)		✓	✓														✓	✓			✓	✓			✓	✓		

✓ means that the parameter indicated in the first row of the table has been collected.

### 3.3 Sleep hygiene: result and discussion

Sleep is a variable of great interest in recent years, due to its probable impact on several problems like impaired cognition, behavioral perturbations, cardiovascular diseases and also obesity (29). In particular, short sleep in childhood is associated with a higher increased risk for obesity and sleep duration has been identified as one of the independent risk factors for weight gain (30). A causal link between sleep quality and weight gain is not established yet, but recent findings suggest an association (31) and several ways through which sleep influences weight status have been proposed: (1) more opportunities for eating, (2) high psychological distress, (3) greater sensitivity to food reward, (4) disinhibited and uncontrolled eating, (5) more energy required to sustain wakefulness, and (6) changes in appetite regulation hormones. In the following chapters, we summarized and discussed shreds of evidence from collected studies, evaluating the influence of sleep duration on eating habits and some adiposity indices.

#### 3.3.1 Sleep restriction and food intake

Several studies suggested that sleep deprivation makes foods more rewarding and might influence dietary choices (32–34).

Hart and colleagues examined the effect of experimental changes in sleep duration in 37 children 8 to 11 years of age, on appetite-regulating hormones, self-reported food intake as well as food reinforcement, and measured weight in two different works (26, 27). Inclusion criteria comprised children > 5th percentile BMI for their age and gender, but no more than 100% overweight, and liking at least 1 of the foods used in the reinforcement paradigm. In the first evaluation (26), children were randomized using a variably sized, stratified (normal weight versus overweight/obese) permuted blocks randomization procedure to increase or decrease the “time between lights out/trying to fall asleep and waking” (TIB) by 1.5 hours/night for 1 week, resulting in a targeted 3-hour TIB difference between conditions. Changes in TIB focused on nocturnal sleep (i.e., naps were not permitted) and were achieved by changing bedtimes, while wake times remained constant. During the sleep increase condition, children reported consuming an average of 134 kcal/day less (P<0,05), and displayed lower fasting morning leptin values (P <0.05) while there were no differences in fasting ghrelin. In addition, measured weights were 0.22 kg lower during the increased sleep than the decreased sleep condition (P< 0,001) (26). The control of leptin in the daytime blood is regulated by multiple factors such as sex, nutrition and fasting, sleep and endocrine alterations (35) and numerous evidence has identified high serum leptin concentrations at baseline as predictors of BMI and greater fat mass over time in children at risk of obesity in adulthood (36–38). If the hours of sleep are prolonged, until at least the recommended levels are reached, the consequent reduction in leptin levels could indirectly affect the risk of developing obesity or being overweight. In the same sample described above (26), the authors (27) examined whether the same above-mentioned experimental changes in children’s sleep lead to changes in reported consumption of high-energy snacks and sugar-sweetened beverages with a 24-h recall. The results showed consuming 35 more calories per day from sugary drinks in the sleep-restricted condition compared to the increased-sleep condition. Interestingly, reduced sleep achieved by delaying bedtime led to increased evening calorie intake and sugary drinks: although no differences were observed in reported intake earlier in the day, from 8pm onward, children reported consuming 132 more calories during the sleep-decrease condition compared to the sleep-increase condition, p< 0.001. Sleep restriction might influence dietary choices as suggested also by Simon et al. (22) who evaluated the effect of a 3-week sleep restriction protocol on adolescents’ subjective hunger and perceived appeal of sweet/dessert foods versus other foods and the influence of sleep restriction on dietary intake. Sleep restriction was considered as 6.5 hours in bed while healthy sleep duration consisted of 10 hours in bed. 31 adolescents aged 14–17 years, without obesity, were enrolled and randomized to two groups (22). Participants were shown food images (42 sweets and 42 non-sweets including fruits and vegetables) and asked to rate how appetizing each image looked on a scale of 1 to 4; adolescents rated their hunger on a 4-point scale from “not hungry” to “very hungry” immediately following food attractiveness ratings. Images of sweets/desserts were rated more appealing in the group with a sleep restriction (t = 2.07, p = .049), but the effect of sleep manipulation was non-significant for self-reported hunger and the appeal of non-sweet foods (22). Morrison et al. (28) provide an experimental manipulation on sleep duration in children to evaluate the influence on energy intake and eating behavior. 105 children (mean age 10.3y) were randomly assigned to begin with sleep restriction or extension: children were asked to go to bed 1 hour earlier (sleep extension condition) and 1 h later (sleep restriction condition) than their usual bedtime for 7 consecutive nights, separated by a 1-wk washout (28). Dietary intake was collected by trained research staff using two 24-h multiple pass diet recalls on days 3 and 8 of each intervention week with the child and parent present and the foods children consumed when sleep deprived were assessed by applying 2 food classification systems: noncore (lower nutrition values like ultra-processed foods, energy-dense foods) or core (healthy foods). Parents were asked to complete a version of the Child Eating Behavior Questionnaire (CEBQ) (39) validated for assessing short-term changes in eating behavior. Differently to studies conducted by Hart (26, 27) authors didn’t find a significant difference in total calorie intake between sleep conditions, although children consumed significantly more carbohydrates as grams, percentage of calories, and total sugars during sleep restriction (28). In addition, during sleep restriction, there was a significantly greater daily intake of calories from energy-dense foods and ultra-processed foods each day. The authors (28) then asked whether the observed relationship between sleep restriction and energy intake or eating behaviors was moderated by weight status, taking into account that 23 children were identified as overweight, and 16% were affected by obesity; these analyses showed that sleep deprivation tended to influence the total energy intake more in the participants with overweight and obesity (28). In the context of CO, Moreno-Frias et al. (25) evaluated the effect of sleep restriction on improving weight loss in adolescents affected by obesity under a caloric-restriction diet. Authors tested the hypothesis that adolescents with obesity under a restricted diet, adding the recommendation of sleep extension, improve weight loss (25). The effects of cortisol, melatonin secretion, and inflammation markers were also evaluated (**Table 2**). 52 adolescents with obesity according to the International Tables of Cole et al. (40) were enrolled. All participants were submitted to dietary restriction that lasted 4 weeks with a caloric restriction of 500 kcal from their previous intake and they were randomly allocated to an intervention group extending sleep duration to 5 minutes each night Monday through Friday, to reach the target of 1 hour for a week and compared with a control group in which participants continued with their usual duration of sleep (25). Sleeping habits were assessed via a sleep diary (25). A sample of venous blood was drawn after a 12-h overnight fast to measure fasting glucose, insulin, cholesterol, triglycerides, leptin, HOMA-index, 6-Sulfatoxymelatonin (ng/mL), cortisol, IL-6 and TNF-alfa. Comparing deltas between both groups of study, Authors found a decrease in weight (p < 0.04) and waist circumference (p < 0.0009) significantly greater in the group with sleep extension (25). No other significant change was found between groups in anthropometric, metabolic, hormone, or proinflammatory parameters but a favorable effect in the sleep extension group was decreased insulin levels, specified a reduction in insulin resistance (25). It is important to notice that not only ponderal status but also individual chronotype may determine differences: evidence show that subjects with a later circadian predisposition (“night owls”) tend to have poorer quality diets than those with an earlier circadian predisposition (“morning larks”) (41, 42). In the study conducted by Beebe et al. (23) authors performed sleep manipulation in two different groups of adolescents aged 14 to 17 years old, divided according to their sleep preference (“night owls” versus “morning larks”). Adolescents changed their bedtimes to match two five-night sleep conditions: sleep restriction corresponding to 6.5 hours in bed versus healthy sleep characterized by 10 hours in bed (23). The total calorie intake of adolescents who entered the study as night owls was not affected by the sleep manipulation but, starting in the evening hours, the morning larks ate less under healthy sleep conditions, suggesting that an “early to bed” approach can lengthen your sleep at night owls without impact on eating behaviors. Instead, the dietary benefit appears more evident among adolescents who are more accustomed to going to sleep earlier, therefore sleep hygiene is a habit that must be established early on. Unfortunately, adolescents with marked obesity were excluded (23) so it was not possible to evaluate the eventual combined effect of chronotype and weight status. However, a recent systematic review that investigates the relationships between sleep outcomes and objective adiposity measures in adolescents suggests that adapting an individual’s schedule to best suit chronotype predisposition and improving sleep hygiene could reduce adiposity and obesity in adolescents (12).

#### 3.3.2 Sleep restriction and anthropometric parameters

Growing evidence has suggested that short sleep duration is associated with increased adiposity and cardiovascular risk markers in youth (43, 44). Carson et al. (24) examined the relationships between sleep duration, sedentary time, physical activity levels and health indicators (**Table 2**) in a cohort of 4169 subjects aged 6-17 years old. A subgroup of 1242 participants also provided a fasting blood sample to insulin to measure lipid profile (total cholesterol, LDL, HDL) triglycerides and C-reactive protein (PCR) (**Table 2**). Sedentary time and physical activity levels were characterize as follows: sedentary time was defined as <100 counts per minute (cpm),light-intensity-physical activity (LPA) as 100–1499 cpm, and moderate-to-vigorous physical activity (MVPA) as ≥1500 cpm (24). Minutes per day of sedentary time, LPA, and MVPA were calculated. Sleep was assessed as part of the in-home health interview (24). The composition of movement behaviors was entered into linear regression models via an isometric log-ratio transformation and authors found interesting results: Time spent in sleep instead to other movement behaviors was negatively associated with BMIz (sleep = –0.93; p = 0.002), log waist circumference (sleep = –0.11; p = 0.001), log systolic blood pressure (sleep = –0.04; p = 0.027), and log behavioral strengths and difficulties (sleep = –0.48; p = 0.001) (24). A similar result was shown by Reis (45) and colleagues who used isotemporal substitution models to examine the estimated effects of substituting time spent on one behavior with an equal amount of time spent on another behavior, while keeping the total time constant while removing the behavior of interest (screen time) from the model. Replacing screen time with sleep time was significant for BMI, waist circumference, systolic blood pressure, fat percentage, and leptin (45). Bodega et al. (19) performed a cluster analysis on lifestyle variables on 1183 adolescents with a mean age of 12.5 years old to identify the association with obesity and they notably found that the least favorable anthropometric profile and the highest prevalence of obesity and overweight between groups of subjects enrolled was characterized by the highest number of steps and the lowest sleep duration, underlining again the role of sleep independent of physical activity level; Authors collected lifestyle variables like diet, physical activity and sleep (**Table 2**). Four lifestyle clusters were pointed out (19): participants in Cluster 1 (C1) showed the unhealthiest profile with the highest screen time scores and the lowest scores in a healthy diet. Participants in Cluster 2 (C2) only scored above average in sleep time and presented the lowest step counts. Participants in Cluster 3 (C3) presented the lowest sleep time score but the highest step counts score and finally, Cluster 4 (C4) showed the healthiest lifestyle profile with scores above average in healthy diet, step counts and sleep time, with the lowest score in leisure screen time (19). Interestingly, participants in C3 had the highest prevalence of overweight/obesity (31.4%) and C4 the lowest (23.1%) and adjusted models show that adolescents in C1, C2, and C3 showed a higher prevalence of overweight/obesity than the cluster with the healthiest lifestyle profile (C4) (19). This study revealed the cluster characterized by shorter sleepers had the most unfavorable anthropometric profile and the highest prevalence of overweight/obesity or central obesity (19). In the same sample, Martin-Gomez and colleagues (20) performed a seven-day accelerometery to characterize sleep duration and its possible cross-sectional and longitudinal associations with adiposity markers in adolescence. Sleep duration was subdivided into three categories based on sleep recommendations in adolescence and participants with a mean sleep duration of 8–10 h per day were considered the reference sleep duration group (20); the remaining groups included participants with very short sleep duration (<7 h/day) and short sleep duration (7–8 h/day) (20). Unfortunately, only 33.7% of adolescents met sleep recommendations, and each decrease in hours of sleep per day was associated with an adjusted increase in BMIz of 0.11 [95% confidence interval (CI): 0.03–0.19] at baseline, 0.12 (95% CI: 0.04–0.19) at first follow-up, and 0.05 (95% CI: −0.02–0.12) at the second follow-up (20); therefore, meeting sleep recommendations at all time points assessed during adolescence was associated with the healthiest adiposity outcomes at approximately 16 years of age, while subjects who never met sleep recommendations showed the more adverse association, with a cumulative dose-response effect (20). This study (20) highlighted the link between insufficient sleep and adverse markers of adiposity, regardless of energy intake and physical activity levels, indicating the main importance of sleep, in agreement with what was found by Bodega and colleagues (19) where, despite the high number of daily steps, the children who slept less had the worst anthropometric profile. Combining this result with findings of Beebe et al. (23) the importance of correct and constant sleep hygiene takes on a relevant role in the prevention of childhood overweight and obesity.

#### 3.3.3 Sleep and eating habits

Two studies (18, 21) were included in this systematic search although they did not consider sleep habits because they evaluated nutritional aspects/eating habits that may be related to sleep habits in children. Several studies investigated the relationship between poor sleep and eating habits, especially in adolescents, considering both dietary choices and eating behavior (22, 26, 27, 46–48). It is believed that among the different synchronization signals for the human circadian system, food intake during the day is one of the main ones (49). Unhealthy eating behaviors like skipping breakfasts, shifting the food intake to a later time in the day (50) and irregular meal timing (51) lead to a deterioration in the quality of sleep, which in turn could worsen daily habits, creating a vicious circle that significantly contributes to the development of obesity or the ineffectiveness of excessive weight treatments. These findings may help explain the link between shortened sleep and increased obesity risk in adolescents. One of the worst eating habits common in children and adolescents is skipping breakfast: a link between obesity and skipping breakfast is well established (52) and sleep could play a role, meaning children who sleep poorly are more at risk of avoiding this meal (53). Two studies (18, 21) were included in this systematic research although they did not consider sleep habits because they evaluated nutritional aspects/eating habits that may be related to sleep habits in children. A recent systematic review demonstrates once again that skipping breakfast is associated with a high prevalence of overweight and obesity (54) and the combined study conducted by Donin et al. (21) highlighted the importance of breakfast during childhood; authors investigated the associations between breakfast type and frequency and risk markers for type 2 diabetes in a cohort of 4,116 primary school children aged 9–10 years (**Table 2**) (21). All participants were asked to complete a questionnaire that included a question on whether they usually ate breakfast in the morning with four options: every day, most days, some days, or not usually (21). Participants were also interviewed by a research nutritionist with a single structured 24-hour recall, in order to categorized breakfast meals type and contents (21). The results showed that 26% of children reported not eating breakfast every day and specifically this percentage included 11% of children who reported eating breakfast most days, 9% on some days and 6% of not consuming it habitually (21). Authors reported that insulin resistance, HbA1c, glucose, triglyceride, C-reactive protein, urate, systolic blood pressure, fat mass index, and sum of skinfolds were all lower and HDL cholesterol higher among children who reported eating breakfast every day and results were not confounded by socioeconomic status and physical activity (21). To evaluate the extent to which these differences in risk markers were mediated by the association between breakfast consumption and adiposity, analyses were repeated with adjustment for fat mass index and sum of skinfolds found the differences in insulin resistance, HbA1c, glucose, and urate were still present (21). In addition, fasting insulin levels and insulin resistance were considerably lower among children eating high fibre cereal compared with other breakfast categories (21). A recent study conducted by Kosti et al. (55) shows an inverse association between sleep duration and weight status independently of breakfast habit, but the co-influence of adequate sleep duration appears to have greater synergy against CO. Skipping breakfast could negatively influence diet for the entire day, also disrupting subsequent eating opportunities. If a child skips breakfast, he will be hungrier during the morning snack, requiring him to consume a quantitatively abundant and qualitatively poor snack, rich in simple sugars, fats and with a high caloric density and also the daily frequency of the meal, the occasions and times of the meals, and eating patterns can influence total energy balance (56, 57).

In the second study collected, Jaeger et al. (18) evaluated the effect of meal timing on CO analyzing data from the Childhood Obesity Project Trial, which include 1678 healthy, full-term infants at the follow-up visits performed during 2005 to 2012 at the ages of 3, 4, 5, 6, and 8 years (18). Authors described the “eating occasion” (EO), any occasion where food or beverages are consumed (18). Predefined categories with typical country-specific time slots were used according to the following EOs: breakfast, lunch, and supper for meals as well as morning, afternoon, and evening for snacks (18). Meal timing was analyzed by compositional data analysis (58, 59). First of all, Authors found that children who are overweight consumed more energy than children with normal weights with a different intake distribution during the day: children consumed higher intakes at lunch and fewer intakes of snacks. No statistically significant differences in weight status were seen for intakes with any EO (18). Most energy intakes were consumed at lunch, followed by snacks, supper, and breakfast. This allows us to speculate on the effect of skipping breakfast: a child who skips breakfast arrives at lunch hungrier and consequently introduces a greater energy intake and this can lead to an increased risk of developing overweight and obesity and increase in blood-chemical markers of adiposity, as noted by Donin (21). Therefore, if sufficient sleep duration is associated with healthier eating habits, while inadequate sleep patterns (for example, sleep restriction) are associated with skipping breakfast and greater consumption of fast food and sweets/candy (20, 27, 28, 60–64), sleep hygiene plays an important role in the context of CO prevention and unhealthy sleep patterns may contribute to the failure of dietary interventions.

## 4 Strength and limitations

The risk of BIAS assessment reported an average weak quality of the included studies. Weak studies were included in this review and study quality was generally limited by participants selection (see **Supplementary Table 1**, column “selection bias”). Another limitation is that the studies collected were largely heterogeneous in terms of length, sample size and characteristics, and particularly hours of sleep manipulated protocols. Furthermore, not all parameters related to the outcome assessed (anthropometric parameters, hormone levels, proinflammatory indices) were evaluated by all the studies analyzed, as particularly evident in **Table 2**.

## 5 Conclusion

Sleep hygiene is a modifiable variable of great interest in the management of CO, despite is often eclipsed by the focus on diet and physical activity.

**Figure 2:**
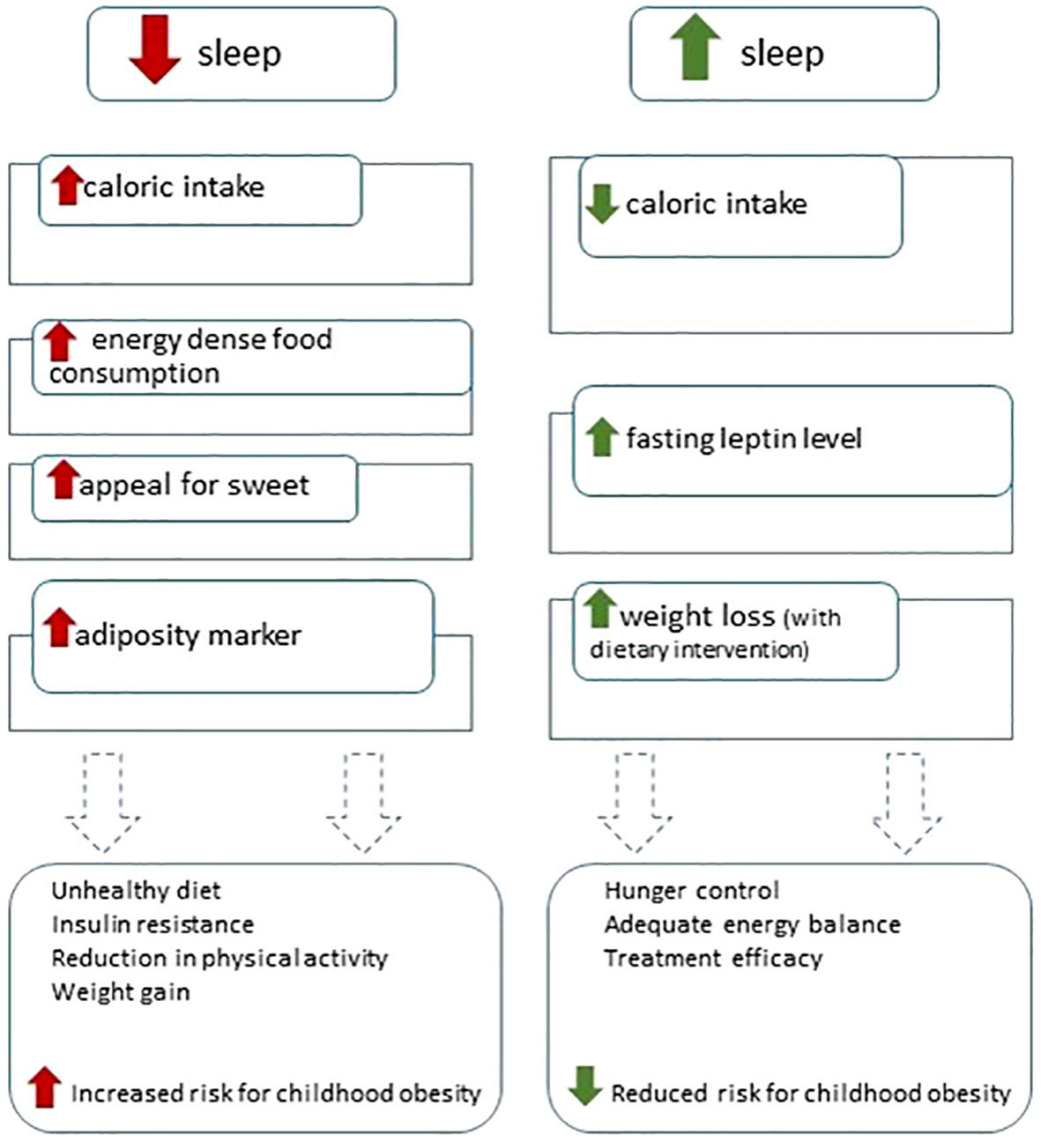
Summary of sleep restriction and sleep extension on childhood obesity risk.

The results of this systematic review instead suggest how poor sleep hygiene can negatively influence eating habits towards unfavorable trajectories and sometimes influence the positive effects of diet and physical activity, significantly contributing to weight gain and exacerbating complications metabolic disorders related to CO. Main results are summarized in **Figure 2**.

However, more rigorous and detailed study designs with validated and standardized measures of sleep duration and manipulation are needed in order to clarify the role of sleep hygiene as a potential weapon to fight CO.

## 6 Recommendations

Although it is not possible to define a completely clear outcome, this review may suggest some useful clinical recommendations for pediatricians:

1. Establishing adequate sleep hygiene can impact long-term health. Parents or caregivers should be educated to establish the child’s sleep habits to ensure correct sleep duration.
2. Investigating sleep preferences and chronotype could help early identify children at increased risk of developing obesity or overweight.
3. Investigating and possibly acting on sleep preferences and chronotype could help make weight loss treatments more effective in children with obesity or overweight.

## Data Availability

The raw data supporting the conclusions of this article will be made available by the authors, without undue reservation.
