# Supplementary material for: Preventing and treating childhood obesity by sleeping better: a systematic review
Source: Front Endocrinol (Lausanne). 2024 Sep 19;15:1426021. doi: 10.3389/fendo.2024.1426021 (PMC11446760; doi:10.3389/fendo.2024.1426021)
Supplement: Supplementary file 1 [file Table1.pdf]

Supplementary Table 1: study quality of final studies, assessed by Effective Public Health Practice Project Quality Assessment Tool For Quantitative Studies

N/A, not applicable; Strong = no weak ratings; Moderate = 1 weak rating; Weak = ≥2 weak rating

Reference	Component Ratings						Global Rating
	Selection Bias	Study Design	Confounders	Blinding	Data Collection Method	Withdrawals & Dropouts	
Jaeger V. et al, 2022 (21)	3	1	1	3	1	3	3 WEAK
Bodega P et al., 2023 (22)	1	1	1	3	1	3	3 WEAK
Martínez-Gómez J et al., 2023 (23)	1	1	3	1	1	3	3 WEAK
Donin AS et al, 2014 (24)	2	1	1	3	1	3	3 WEAK
Simon SL et al., 2015 (25)	3	1	1	3	1	1	3 WEAK
Beebe D.W. et al, 2015 (26)	3	1	1	3	1	1	3 WEAK
Carson V, et al., 2016 (27)	1	1	1	3	1	3	3 WEAK
Moreno-Frías C et al., 2020 (28)	3	1	1	3	1	3	3 WEAK
Hart C. et al., 2013 (29)	3	1	3	3	2	1	3 WEAK
Hart C. et al., 2022 (30)	3	1	3	3	2	1	3 WEAK
Morrison S et al, 2023 (31)	3	1	1	3	1	1	3 WEAK
